# Motor polyradiculoneuropathy as an unusual presentation of neurobrucellosis: a case report and literature review

**DOI:** 10.1186/s12879-024-09365-2

**Published:** 2024-05-14

**Authors:** Ahmad Alikhani, Noushin Ahmadi, Mehran Frouzanian, Amirsaleh Abdollahi

**Affiliations:** 1 Infectious Diseases Department and Antimicrobial Resistance Research Center and Transmissible Diseases Institute, Mazandaran University of Medical Sciences, Sari, Iran; 2 Student Research Committee, School of Medicine, Mazandaran University of Medical Sciences, Sari, Iran

**Keywords:** Neurobrucellosis, EMG-NCV tests, Polyradiculoneuropathy, Antibiotic therapy, Intravenous immunoglobulin therapy, Zoonotic disease, Gait disorder, Lower extremity weakness, Diplopia, Blurred vision

## Abstract

Brucellosis, a zoonotic disease caused by *Brucella* species, poses a significant global health concern. Among its diverse clinical manifestations, neurobrucellosis remains an infrequent yet debilitating complication. Here, we present a rare case of neurobrucellosis with unusual presentations in a 45-year-old woman. The patient’s clinical course included progressive lower extremity weakness, muscle wasting, and double vision, prompting a comprehensive diagnostic evaluation. Notable findings included polyneuropathy, elevated brucella agglutination titers in both cerebrospinal fluid and blood, abnormal EMG-NCV tests, and resolving symptoms with antibiotic therapy. The clinical presentation, diagnostic challenges, and differentiation from other neurological conditions are discussed. This case underscores the importance of considering neurobrucellosis in regions where brucellosis is prevalent and highlights this rare neurological complication’s distinctive clinical and radiological features. Early recognition and appropriate treatment are crucial to mitigate the significant morbidity associated with neurobrucellosis.

## Introduction

Brucellosis, caused by *Brucella* species, is an infectious ailment recognized by various names such as remitting, undulant, Mediterranean, Maltese, Crimean, and goat fever. Humans contract it through the consumption of unpasteurized milk and dairy products, undercooked meat, or skin contact with infected livestock [1–3]. Various *Brucella* species, including *Brucella melitensis* (primarily sourced from sheep and goats), Brucella abortus (found in cattle), *Brucella suis* (associated with pigs/hogs), and *Brucella canis* (linked to dogs), can lead to illness in humans [3–5]. While brucellosis in humans is rarely fatal, it can lead to disability [6]. Brucellosis ranks among the most prevalent zoonotic diseases, impacting approximately 500,000 individuals yearly [7]. The combined estimate for the prevalence of brucellosis was 15.53% [8].

Neurobrucellosis, a rare complication of systemic brucellosis, can occur in adult and pediatric cases [9], and can manifest at any stage of the disease. They can present in various clinical presentations such as meningitis, encephalitis, meningoencephalitis, myelitis, radiculopathy, polyneuropathy, stroke, cerebral venous thrombosis, and occasionally psychiatric symptoms [10, 11]. Although the mortality rate is low, patients often experience persistent neurological issues following neurobrucellosis [12]. Studies suggest that around 20% of neurobrucellosis cases result in lasting neurological problems [13]. It is uncommonly considered in cases of meningoencephalitis or polyneuropathy, making it crucial for clinicians to have a high suspicion of it in patients displaying such symptoms, especially in endemic regions, to prevent severe clinical outcomes. In this study, we present a rare case of neurobrucellosis with unusual clinical presentations in a patient admitted to our center.

## Case presentation

A 45-year-old female patient, with no prior medical history, presented to our center after enduring distal pain and weakness in her lower extremities for approximately 10 months. Over this period, the muscle weakness progressed, affecting proximal muscles of upper and lower limbs, and leading to a substantial weight loss of 25–30 kg despite maintaining appetite. Initially dismissive of the limb weakness and pain, the patient sought medical attention six months after symptom onset due to the worsening symptoms and gait impairment. Over the subsequent four months, she underwent multiple medical evaluations and tests, including a lumbar X-ray. Following these initial investigations and due to low serum vitamin D levels, vitamin D and calcium supplements were prescribed, and lumbar MRI were requested for further evaluation. (Table 1)

**Table 1:** Laboratory test on admission and 5 days after admission

Variable	During admission	Day 5	Reference range
Hemoglobin (g/dl)	10.5	10	13.5–17.5
MCV (fl.)	76	81	81–97
White-cell count (per µl)	6,400	4,800	4,500–13,000
Differential count (%)			
Neutrophils	56	38.5	40.0–62.0
Lymphocytes	36	46.6	27.0–40.0
Platelet count (per µl)	222,000	252,000	150,000–400,000
Fasting blood sugar (mg/dL)	111	-	70–100
Urea nitrogen (mg/dl)	18	19	6–24
Creatinine (mg/dl)	0.9	0.9	0.60–1.50
Free T3 (pg/mL)	2.9	-	2.3–4.1
T4 (µg/dL)	8.56	-	5–12
TSH (mIU/L)	1.27	-	0.4-4
Rheumatoid factor (IU/mL)	4	-	0–20
Erythrocyte sedimentation rate (mm/hr)	9	8	0–22
C-reactive protein (mg/liter)	4	5	0–10
Alanine aminotransferase (U/liter)	-	10	10–40
Aspartate aminotransferase (U/liter)	-	13	10–40
Alkaline phosphatase (U/liter)	-	100	44–196
Wright	1/80	1/80	
2ME	1/80	1/40	
Coombs Wright	1/320	1/80	

Upon referral to an infectious disease specialist, the patient’s history of local dairy consumption and positive serologic test for brucellosis prompted treatment with rifampin and doxycycline. However, the patient’s condition deteriorated significantly five days after starting this treatment. She experienced severe gait disorder, lower extremity weakness, diplopia, and blurred vision that had gradually worsened over two weeks. Subsequently, she presented to our center for further assessment.

Upon admission, the patient was unable to stand even with assistance and exhibited diplopia. Cranial nerve examination revealed no abnormalities, except for the II, III, and IV cranial nerves, which could not be thoroughly examined due to the presence of diplopia. The patient tested negative for Kernig and Brudzinski signs. There were no palpable supraclavicular or inguinal lymph nodes. Physical examinations of the breast, axilla, lungs, heart, and abdomen were unremarkable. Muscle strength was reduced in the lower extremities, and deep tendon reflexes of the knee and Achilles were absent. The plantar reflex was non-responsive, and certain reflexes, including biceps, triceps, and brachioradialis, were absent despite normal movement of the upper extremities. Anorectal muscle tone and anal reflex were normal.

Further investigations included normal urinalysis and abdominal and pelvic ultrasound. Chest X-ray and brain CT were also ordered. Due to the patient’s refusal of lumbar puncture, a suspicion of neurobrucellosis led to the initiation of a three-drug regimen (Table 2); ceftriaxone 2 g IV twice daily, rifampin 600 mg PO daily, and doxycycline 100 mg PO twice daily. The ophthalmology consultation did not reveal any ocular pathology, and the neurologist ordered brain MRI and EMG-NCV tests. The patient’s brain MRI was unremarkable, but EMG-NCV showed sensory and motor polyneuropathy. Consequently, intravenous immunoglobulin (IVIG) therapy was initiated at a daily dose of 25 g. After five days, the patient consented to lumbar puncture, confirming the diagnosis of brucellosis. Co-trimoxazole 960 mg PO three times daily was added to her treatment regimen, and IVIG therapy continued for seven days. Following a 3-day course of IVIG treatment, the neuropathy symptoms showed significant improvement. By the seventh day, there was a notable enhancement in limb strength, particularly in the upper limbs, reaching a 2-point improvement. After undergoing three weeks of intravenous therapy, the patient transitioned to oral medication. Despite disagreement regarding the necessity of a second CSF examination, the patient was discharged with a prescription for doxycycline, rifampin, and cotrimoxazole. Upon discharge, the patient could walk with the aid of a walker. However, within a month, a slight limp persisted, and by the third-month post-discharge, all symptoms had resolved completely.

**Table 2:** CSF results of patient after lumbar puncture

Variable	Patient	Normal range
WBC	100	0–5 (upto 30 in neonates)
Neutrophils	18	50–60%
lymphocytes	82	20 − 0%
RBC	500	Nil
Protein	110	15–40 mg/dL
Glucose	55	50–80 mg/dL (two-thirds of blood glucose)
Blood glucose	87	Up to 126
Wright	1/80	
Appearance	Slightly turbid	Clear
Pressure	195	90–180 mmHg

## Discussion

Brucellosis is widely spread globally, with more than half a million reported human cases annually [14, 15]. Countries like Kenya, Yemen, Syria, Greece, and Eritrea have experienced high rates of brucellosis. The situation of brucellosis has shown signs of improvement in many epidemic regions. However, new areas with high occurrences of this disease continue to emerge, particularly in Africa and the Middle East, where the incidence of the disease varies [16]. Brucellosis is linked to various neurological complications collectively known as neurobrucellosis, which is an uncommon condition, and only a few cases have been reported globally [17–21]. Our patient exhibited muscle weakness, polyneuropathy, and inability to walk, which are often not regarded as indicative of a brucella infection by many physicians. While the diagnosis of neurobrucellosis can typically be confirmed through classical clinical signs, radiological examinations, and serological tests, patients might not always display typical symptoms, as observed in our case. Hence, in regions where the disease is prevalent, clinicians should maintain a high level of suspicion if patients do not show improvement with standard treatment. Additionally, the lack of awareness among healthcare professionals and limited access to advanced laboratory facilities can lead to misdiagnosis.

The frequent manifestations of neurobrucellosis include meningitis or meningoencephalitis. Typically, it starts with a sudden headache, vomiting, and altered mental state, which can progress to unconsciousness, with or without seizures [22]. Additionally, brucellosis can lead to several central nervous system issues such as inflammation of cerebral blood vessels, abscesses in the brain or epidural space, strokes, and cerebellar ataxia. Peripheral nerve problems may include nerve damage or radiculopathy, Guillain-Barré syndrome, and a syndrome resembling poliomyelitis [13]. Nevertheless, the patient exhibited no indications of seizures, brain hemorrhage, stroke, or focal neurological impairments. Instead, the observed symptoms were consistent with radiculopathy and muscular weakness.

In only 7% of neurobrucellosis cases, the peripheral nervous system is affected. Remarkably, our case falls within this rare category, adding to its unique and intriguing nature. Previous case studies have detailed polyradiculoneuropathies, manifesting as acute, subacute, or chronic forms [23]. Our patient’s condition aligns with chronic motor polyradiculopathy. Interestingly, some of these cases exhibit sensory deficits or resemble Guillain-Barré syndrome [23, 24]. In a prior case study conducted by Abuzinadah and colleagues, a comparable case was described as a subacute motor polyradiculopathy. The patient exhibited gradual bilateral lower limb weakness over three weeks, eventually leading to loss of mobility within seven weeks. Brucella was isolated from the cerebrospinal fluid after a two-week incubation period, and high antibody titers were detected in the patient’s serum [23]. In another study led by Alanazi and colleagues, a 56-year-old man initially diagnosed with Guillain-Barré syndrome experienced worsening symptoms despite appropriate treatment. Following plasma exchange and antibiotics, his condition improved temporarily, only to relapse, raising suspicion of chronic inflammatory demyelinating polyneuropathy, and treatment with IVIG resulted in substantial improvement. Upon further investigation, he was diagnosed with brucellosis [24]. This highlights the importance of recognizing GBS-like symptoms in regions where brucellosis is prevalent, prompting clinicians to consider the possibility of brucellosis in their diagnosis.

While there are no established criteria for diagnosing neurobrucellosis [25], certain articles have suggested several methods for its diagnosis. These methods include the presence of symptoms aligning with neurobrucellosis, isolating brucella from cerebrospinal fluid (CSF) or detecting a positive brucella agglutination titer in CSF, observing lymphocytosis, elevated protein, and decreased glucose levels in CSF, or identifying specific diagnostic indicators in cranial imaging such as magnetic resonance imaging or computed tomography (MRI or CT) [13, 26–28]. Neurobrucellosis does not present a distinct clinical profile or specific CSF characteristics. Imaging observations of neurobrucellosis fall into four categories: normal, inflammatory (indicated by granulomas and enhanced meninges, perivascular spaces, or lumbar nerve roots), alterations in white matter, and vascular changes [29]. We suspected neurobrucellosis based on the patient’s clinical symptoms, geographic correlation, high brucella agglutination test titers in both cerebrospinal fluid and blood, symptom resolution following treatment, and the exclusion of other common causes.

In Iran, one differential diagnosis often confused with brucellosis is tuberculosis, as both chronic granulomatous infectious diseases are prevalent here [30, 31]. Neurobrucellosis and tuberculosis exhibit significant similarities in clinical symptoms, lab results, and neuroimaging findings. However, deep grey matter involvement and widespread white matter lesions seen in neuroimaging, resembling demyelinating disorders, appear to be distinctive to brucellosis [32]. There is a noticeable similarity in the clinical symptoms and laboratory findings of brucellosis and tuberculosis [33]. It is crucial to thoroughly eliminate the possibility of tuberculosis in any suspected or confirmed brucellosis cases before starting antibiotic treatment.

Due to the challenging nature of treating brucellosis and the likelihood of experiencing relapses, it is crucial to provide an extended course of treatment [27]. This treatment approach should involve a combination of antibiotics that can easily penetrate the cell wall and effectively reach the central nervous system [27, 34]. Neurobrucellosis is treated with 3 to 6 months of combination therapy comprising doxycycline, rifampicin, and ceftriaxone or trimethoprim-sulfamethoxazole [35], similar to the treatment administered to our patient. For patients allergic to cephalosporins, quinolones are recommended, which are considered to be effective in treating brucellosis [36, 37]. In complicated situations such as meningitis or endocarditis, streptomycin or gentamicin is administered in the initial 14 days of treatment, in addition to the previously mentioned regimen. Timely and proper treatment results in a positive prognosis, with a less than 1% fatality rate for such complex cases [17, 38]. Our patient experienced a highly positive outcome following the prescribed therapy. Initially relying on a walker, a slight limp endured for a month, and by the third month after discharge, all symptoms completely disappeared.

## Conclusion

The present study underscores the significance of considering neurobrucellosis as a potential diagnosis when evaluating muscle weakness and radiculopathy, especially in regions where the disease is prevalent. A comprehensive patient history, precise clinical examination, positive serology in blood or cerebrospinal fluid, imaging results, or cerebrospinal fluid analysis can contribute to establishing a conclusive diagnosis.

## Data Availability

The datasets generated and/or analysed during the current study are not publicly available due to our team’s privacy concerns but are available from the corresponding author on reasonable request.
